# Near-Real-Time Epileptic Seizure Detection with Reduced EEG Electrodes: A BiLSTM-Wavelet Approach on the EPILEPSIAE Dataset

**DOI:** 10.3390/brainsci16010119

**Published:** 2026-01-22

**Authors:** Kiyan Afsari, May El Barachi, Christian Ritz

**Affiliations:** 1 School of Engineering, University of Wollongong in Dubai, Dubai P.O. Box 20183, United Arab Emirates; 2 School of Computer Science, University of Wollongong in Dubai, Dubai P.O. Box 20183, United Arab Emirates; maielbarachi@uowdubai.ac.ae; 3 School of Electrical, Computer and Telecommunications Engineering, University of Wollongong, Wollongong 2522, Australia; critz@uow.edu.au

**Keywords:** EEG, seizure detection, epilepsy

## Abstract

**Background and Objectives:** Epilepsy is a chronic neurological disorder characterized by recurrent seizures caused by abnormal brain activity. Reliable near-real-time seizure detection is essential for preventing injuries, enabling early interventions, and improving the quality of life for patients with drug-resistant epilepsy. This study presents a near-real-time epileptic seizure detection framework designed for low-latency operation, focusing on improving both clinical reliability and patient comfort through electrode reduction. **Method:** The framework integrates bidirectional long short-term memory (BiLSTM) networks with wavelet-based feature extraction using Electroencephalogram (EEG) recordings from the EPILEPSIAE dataset. EEG signals from 161 patients comprising 1032 seizures were analyzed. Wavelet features were combined with raw EEG data to enhance temporal and spectral representation. Furthermore, electrode reduction experiments were conducted to determine the minimum number of strategically positioned electrodes required to maintain performance. **Results:** The optimized BiLSTM model achieved 86.9% accuracy, 86.1% recall, and an average detection delay of 1.05 s, with a total processing time of 0.065 s per 0.5 s EEG window. Results demonstrated that reliable detection is achievable with as few as six electrodes, maintaining comparable performance to the full configuration. **Conclusions:** These findings demonstrate that the proposed BiLSTM-wavelet approach provides a clinically viable, computationally efficient, and wearable-friendly solution for near-real-time epileptic seizure detection using reduced EEG channels.

## 1. Introduction

Epilepsy is a chronic neurological disorder that affects more than 50 million people worldwide [1]. It is characterized by sudden, recurrent surges of abnormal brain activity that manifest as convulsive seizures and episodes of impaired awareness, memory, or consciousness [2]. Despite advances in pharmacological and surgical treatments, nearly 30% of patients remain drug-resistant and continue to experience unpredictable seizures [3]. For such patients, timely and reliable seizure detection is critical to improve quality of life and reduce the risks of injury, hospitalization, or mortality associated with prolonged seizures [4].

Electroencephalography (EEG) is the gold standard for monitoring brain activity, providing detailed insights into electrical patterns that accompany seizure onset and progression [5]. Neurologists can manually review EEG recordings to identify seizures; however, this approach is labor-intensive, subjective, and impractical for real-time monitoring in clinical or home settings [6]. Automated seizure detection systems are therefore essential to provide continuous, accurate, and scalable support for clinical decision-making and patient safety.

Seizures are commonly categorized as focal, generalized, or unknown in onset [7]. Their variability across patients and within the same patient poses a significant challenge for automated detection [8]. Traditional seizure detection methods have relied on handcrafted EEG features combined with machine learning classifiers such as support vector machines (SVMs) and artificial neural networks (ANNs) [9,10]. While these methods have demonstrated promise, they often require extensive preprocessing and are limited in their ability to generalize across large and diverse datasets.

Deep learning approaches have recently gained momentum due to their capacity to automatically extract complex spatiotemporal features from EEG signals. Convolutional neural networks (CNNs) [11], recurrent neural networks (RNNs) [12], and their variants have been successfully applied to retrospective seizure detection tasks [13]. However, most existing works focus on offline detection, analyzing data after seizure occurrence, rather than supporting real-time detection, which is essential for proactive interventions. In addition, many approaches depend on high-density EEG setups with more than 32 electrodes, which, while improving accuracy, reduce patient comfort and limit portability.

This study presents a comprehensive evaluation framework for near-real-time seizure detection that leverages bidirectional long short-term memory (BiLSTM) networks combined with wavelet-based frequency-domain features. In this study, near-real-time detection refers to the ability to process incoming EEG windows and produce a seizure detection decision within a latency shorter than the analysis window duration, without requiring access to future samples. While BiLSTM and wavelet-based features are well-established, our work focuses on the systematic evaluation of hybrid feature-network combinations under practical constraints, including reduced electrode montages and real-time execution. Different network configurations within the BiLSTM frameworks were tested, optimizing parameters such as layer depth and hidden units, to identify robust setups for seizure classification. Exploration of entirely new network architectures is planned as future work. This study therefore contributes by providing a detailed assessment of hybrid feature-network strategies on the EPILEPSIAE dataset, highlighting practical performance considerations for real-world applications. The model was trained and evaluated on the EPILEPSIAE dataset, which is the largest publicly available epilepsy dataset, comprising EEG recordings from 161 patients and more than 1000 labeled seizures collected across four European hospitals [14]. In addition to near-real-time performance analysis, electrode reduction strategies are investigated to enable comfortable, low-cost wearable applications. An outlier-aware retraining scheme is also introduced to improve detection of problematic seizures that are often missed by generalized models. The main contributions of this work are as follows:

1. Design and evaluation of a BiLSTM-based deep learning model for near-real-time seizure detection with an average processing latency below the analysis window duration.
2. Development of a feature integration strategy combining raw EEG signals with wavelet transforms to enhance classification robustness.
3. Exploration of input reduction methods demonstrating reliable detection with as few as six electrodes, improving wearability.
4. Comparative analysis between the EPILEPSIAE dataset [14] and the MIT dataset [15] to assess generalizability.

The remainder of this paper is organized as follows: Section 2 reviews related works in EEG-based seizure detection. Section 3 presents the proposed methodology and model design. Section 4 discusses experimental results and analysis. Section 5 concludes this study.

## 2. Literature Review

This section critically examines existing research in automated epileptic seizure detection using EEG signals. It systematically reviews traditional machine learning approaches, the emergence of deep learning, and current advancements and limitations, particularly concerning real-time performance and practical application.

### 2.1. Overview of EEG for Seizure Detection

Electroencephalography (EEG) is the standard technique for monitoring brain activity in patients with epilepsy [5,16]. It provides high temporal resolution and directly measures neuronal electrical activity, which is critical for identifying the rapid and transient changes associated with seizure onset [17]. Characteristic EEG features such as spikes, sharp waves, and rhythmic discharges enable distinction between seizure and non-seizure states [18].

EEG acquisition can be performed using two main modalities. Scalp EEG involves non-invasive electrode placement on the head surface and is widely used in clinical and research contexts due to its accessibility and safety [19]. Intracranial EEG (iEEG), by contrast, requires surgical implantation of electrodes near the epileptogenic zone, offering greater spatial resolution at the expense of invasiveness [20]. In most scalp EEG studies, the international 10–20 system provides a standardized framework for electrode placement across patients [21].

For seizure detection research, EEG is particularly advantageous because it supports continuous long-term monitoring, enables real-time analysis, and reveals seizure-specific electrographic signatures. These properties make EEG the primary modality for developing automated seizure detection algorithms and form the foundation for the machine learning and deep learning methods discussed in subsequent sections.

### 2.2. Traditional Machine Learning Approaches for EEG Seizure Detection

#### 2.2.1. Feature Extraction

Early research in automated seizure detection relied heavily on handcrafted feature extraction from EEG signals [22]. Features were typically derived from three main domains:

- Time-domain features, such as amplitude, variance, standard deviation, zero-crossing rate, and Hjorth parameters, were widely used to capture the statistical properties of EEG activity [23].
- Frequency-domain features, including band power in the delta, theta, alpha, beta, and gamma ranges, were extracted using Fourier or wavelet transforms to characterize rhythmic oscillations associated with seizure activity [24].
- Non-linear and dynamical features, such as entropy measures, fractal dimensions, and chaos-based parameters, were employed to capture the irregularity and complexity of brain dynamics [25].

While effective in controlled settings, these handcrafted features required significant domain expertise, were often sensitive to noise and artifacts, and demonstrated poor generalizability across patients due to strong inter and intra-patient variability [26].

#### 2.2.2. Classification Algorithms

The extracted features were typically input to machine learning classifiers, including support vector machines (SVMs), k-nearest neighbours (kNNs), decision trees, and artificial neural networks (ANNs) [27]. These methods provided valuable insights into the feasibility of automated seizure detection and achieved reasonable performance in small-scale studies. However, they exhibited several limitations. Classifier performance was highly dependent on the relevance and quality of the handcrafted features. In addition, many of these algorithms struggled with high-dimensional, non-stationary EEG data, and their ability to model complex spatiotemporal dependencies remained limited.

#### 2.2.3. Early Attempts at Online Detection

Some early works attempted to move beyond retrospective analysis and proposed methods for real-time or online seizure detection [28]. These approaches typically relied on simplified features and lightweight classifiers to meet computational constraints. While they represented important progress, such methods often faced significant trade-offs between speed and accuracy. Many failed to achieve clinically acceptable levels of sensitivity and suffered from high false alarm rates, which limited their practical adoption.

Traditional machine learning approaches laid the groundwork for automated seizure detection by introducing systematic feature extraction and classification frameworks. However, their reliance on handcrafted features, limited capacity for capturing temporal dynamics, and weak generalization across diverse patient populations highlighted the need for more advanced methods. These shortcomings motivated the transition toward deep learning approaches, which are discussed in the following section.

### 2.3. Deep Learning in EEG Seizure Detection: Advancements and Gaps

Deep learning techniques have significantly advanced the field of automated EEG-based seizure detection by enabling models to learn complex spatial and temporal patterns directly from data [11]. These methods have reduced the dependence on handcrafted features and demonstrated improved generalization in many settings.

Convolutional Neural Networks (CNNs) have been widely applied to EEG seizure detection [29]. CNNs can automatically extract hierarchical spatial features from multichannel EEG signals by interpreting them as structured inputs, such as two-dimensional representations or sequential data with spatial correlations [30]. Numerous studies have reported strong performance using CNN architectures for seizure classification and detection tasks [11]. Despite their strengths in spatial feature extraction, CNNs are less effective at modeling long-range temporal dependencies unless combined with additional mechanisms, and most applications still focus on offline rather than real-time detection [29].

Recurrent Neural Networks (RNNs) and their variants, particularly Long Short-Term Memory (LSTM) and Bidirectional LSTM (BiLSTM) networks, are better suited for sequential data because they can capture temporal dependencies and patterns over time [31]. Several works have shown that LSTMs can improve seizure detection by leveraging the sequential structure of EEG signals [32,33]. However, many of these studies also operate in retrospective settings, and relatively few optimize their architectures for real-time deployment with strict latency requirements. Hybrid deep learning models have emerged to combine the strengths of different architectures. Examples include CNN–RNN hybrids that use CNN layers for spatial feature extraction, followed by RNN or LSTM layers for temporal modeling [34]. Other approaches integrate deep learning with traditional feature extraction methods, such as wavelet transforms, to enhance robustness [35]. These hybrid architectures have achieved promising results, but they often increase model complexity and computational demands, which can make real-time implementation challenging.

A key distinction in the literature is between offline and real-time detection. Many high-performing models are evaluated in offline contexts, where the system has access to complete EEG recordings for analysis after seizures occur. Although this setting is useful for benchmarking, it is insufficient for clinical applications that require rapid detection at or near seizure onset. Real-time systems must make predictions with low latency and limited future context, which introduces additional challenges in model design, optimization, and evaluation. While deep learning has led to substantial performance gains in seizure detection, persistent gaps remain. Many approaches are not optimized for real-time operation, rely on complex architectures that are difficult to deploy on wearable devices, or lack systematic validation on large-scale clinical datasets. These limitations highlight the need for models that balance detection accuracy, computational efficiency, and practical applicability.

### 2.4. Addressing Practical Constraints: Wearability, Electrode Reduction, and Data Imbalance

Despite recent algorithmic advances, practical deployment of seizure detection systems faces several important constraints. These include the wearability of EEG setups, the number of electrodes required, the computational burden of deep models, and the imbalance between seizure and non-seizure data.

High-density EEG systems, typically with more than 32 electrodes, are commonly used in research to maximize spatial information and detection accuracy [36,37]. However, such systems are cumbersome, uncomfortable for prolonged use, and expensive, making them unsuitable for daily monitoring. Electrode reduction has therefore become an important research focus [38]. Studies have explored various channel selection and feature selection methods to identify smaller electrode subsets while maintaining acceptable detection performance [39,40]. Existing attempts at electrode reduction have demonstrated performance trade-offs, and systematic evaluations on large-scale clinical datasets remain scarce [39]. In many cases, the reduced channel configurations are also patient-specific, meaning that the optimal set of electrodes differs between individuals, which reduces generalizability and complicates clinical implementation [40]. Computational efficiency is another major consideration for real-time seizure detection. Deep learning models often require significant processing power, which can limit their use on low-power wearable or implantable devices [41]. Efforts to address this issue include model compression, pruning, and lightweight architectures designed to reduce inference time without substantial loss in performance [42,43]. Ensuring near-real-time response is essential for clinical relevance, especially in applications where early detection can enable timely interventions.

Data imbalance poses a further challenge. Seizure events are relatively rare compared to normal brain activity, resulting in datasets with a large skew toward non-seizure segments. This imbalance can bias models toward predicting the majority class, leading to misleading accuracy metrics and reduced sensitivity. To mitigate this problem, studies have employed techniques such as oversampling, undersampling, cost-sensitive learning, focal loss functions, and synthetic data generation using augmentation or generative adversarial networks [44]. Effective handling of class imbalance is essential to achieve reliable seizure detection performance, particularly in real-time scenarios where false negatives carry significant clinical risk.

Together, these factors underline the importance of designing seizure detection systems that are not only accurate but also practical, efficient, and robust to real-world variability. Addressing these constraints is a critical step toward translating deep learning-based seizure detection methods from research environments to clinical and wearable applications.

### 2.5. Identification of Research Gaps and Contributions of This Work

Table 1 summarizes representative EEG-based seizure detection studies in terms of model architecture, detection accuracy, and latency. As shown, existing approaches have demonstrated high detection accuracy using a variety of deep learning and signal processing techniques, including CNN-based architectures, recurrent networks, and wavelet-domain analysis. However, several limitations remain. First, detection latency is often on the order of multiple seconds, which may limit practical near-real-time deployment despite high accuracy. Second, many studies evaluate performance under fixed window lengths or offline conditions without reporting end-to-end computational delay. Third, differences in dataset characteristics and evaluation protocols make direct comparison difficult, particularly with respect to electrode count and real-time feasibility.

The literature reviewed in the preceding sections highlights several persistent limitations in automated seizure detection research. Despite significant advances in both traditional machine learning and deep learning, the following gaps remain:

1. Near-real-time detection limitations: Many existing studies focus on retrospective or offline seizure detection, where algorithms analyze data after seizure occurrence [50]. While useful for post-hoc analysis, such approaches are inadequate for proactive clinical intervention. There is a lack of robust deep learning models explicitly optimized for near-real-time seizure detection with low latency, typically under a second to a few seconds, which is essential for timely clinical response.
2. Wearability and electrode reduction: A majority of deep learning studies employ high-density EEG setups with more than 32 electrodes, which, although beneficial for accuracy, are impractical for long-term monitoring due to patient discomfort, setup complexity, and limited portability. Existing attempts at electrode reduction have demonstrated performance trade-offs, and systematic evaluations on large-scale clinical datasets remain scarce [38,51]. In many cases, the reduced channel configurations are also patient-specific, meaning that the optimal set of electrodes varies between individuals, which limits generalizability and complicates practical implementation [52].
3. Inter and intra-patient variability: Seizure manifestations differ significantly across patients and even within the same patient, leading to challenges in generalization [52]. Current deep learning models often fail to adapt to irregular or outlier seizure patterns, resulting in reduced detection reliability. Few studies have introduced mechanisms for adaptive retraining or outlier-aware detection to address these issues [44,53].
4. Evaluation on large, diverse datasets: Several influential works rely on smaller benchmark datasets such as the MIT scalp EEG database [15], which provide limited representation of real-world clinical variability. Comprehensive evaluations on large-scale datasets, such as EPILEPSIAE, under stringent near-real-time and reduced-electrode conditions, remain underexplored [14].

This study directly addresses these gaps by proposing a BiLSTM-based framework that integrates wavelet-derived frequency-domain features with raw EEG data to enable robust near-real-time seizure detection. The framework is trained and validated on the large-scale EPILEPSIAE dataset comprising recordings from 161 patients and over 1000 seizures [14]. In addition, electrode reduction strategies are systematically evaluated to assess performance under wearable-friendly configurations. An outlier detection and retraining mechanism is incorporated to improve adaptability to irregular seizure patterns. Finally, a comparative analysis with the MIT dataset is presented to demonstrate the generalizability of the proposed approach across different clinical contexts.

## 3. Methodology

The experiments in this study were conducted on the EPILEPSIAE dataset, the largest publicly available epilepsy database, which contains recordings from 161 patients and more than 1000 annotated seizures [14]. The dataset includes continuous EEG signals recorded with 19 to 32 scalp electrodes, supplemented by additional biometric modalities such as electromyography (EMG), electrocardiography (ECG), and, in some cases, electrooculography (EOG) [14]. Data collection took place across four major European hospitals. The surf_30 subset was obtained from Freiburg University Hospital Medical Biometry and Statistics (UKLFR) between 2002 and 2009. The Coimbra and Coimbra10 subsets were acquired at the Hospitais da Universidade de Coimbra (HUC) from 2009 to 2011. The pa_surf subset originates from Paris, France. All EEG recordings follow the international 10–20 system for electrode placement. While electrode availability varied across patients due to the extended data collection period, 19 scalp locations were consistently present across all subsets. The average annotated seizure duration was approximately 60 s.

EEG provides a detailed representation of brain dynamics and can support diverse analyses, including energy distribution, neurological disorder diagnosis, and sleep research. However, extracting meaningful features from EEG often requires extensive preprocessing and careful feature engineering, which are both time-intensive and dataset-specific. Deep learning approaches offer a more direct alternative by learning robust features directly from raw or minimally processed EEG signals. Traditional feed-forward neural networks, however, are limited in their ability to capture temporal dependencies within EEG sequences. Recurrent architectures, particularly Long Short-Term Memory (LSTM) networks, incorporate memory mechanisms that enable effective modeling of sequential and dynamic patterns.

**Figure 1:**
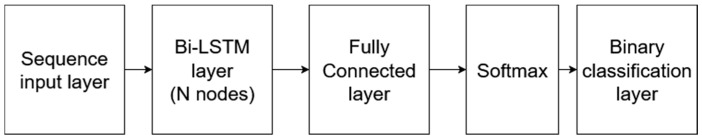
Proposed BiLSTM—wavelet seizure detection framework. Overview of the pipeline including preprocessing, wavelet feature extraction, BiLSTM model, and final seizure detection.

In this work, a baseline BiLSTM model was first employed to establish a generalized seizure detection framework. The model was then further enhanced through hyperparameter optimization and EEG channel reduction to improve both accuracy and practicality for wearable applications. In addition, hybrid deep learning models were evaluated on feature-engineered data, integrating temporal and frequency domain characteristics to assess whether they provided further improvements over the generalized BiLSTM approach. The overall framework is summarized in Figure 1.

Epileptic seizures occur unpredictably and vary in duration, which alters the priorities for seizure detection systems. Unlike conventional classification tasks, where overall accuracy is often emphasized, sensitivity and detection latency are the most critical performance metrics in this domain. Since seizures are relatively rare events, the dataset is inherently imbalanced, with non-seizure periods vastly outnumbering seizure occurrences. This imbalance can produce misleading results. For example, a 30 s seizure within a one-hour recording represents only 0.8% of the data; a classifier could report more than 99% accuracy while entirely failing to detect the seizure.

In seizure detection, metrics beyond accuracy are therefore essential. Recall (sensitivity) is particularly important, as it reflects the proportion of seizures correctly identified. While the ideal goal is to optimize both precision and recall, in practice, higher recall is prioritized. Minimizing false negatives is of greater clinical importance than minimizing false positives, since overlooking a seizure carries far greater medical risk than issuing an occasional false alarm.

Detection delay is a critical metric that reflects the latency between seizure onset and model prediction [12]. Detection delay was computed as the temporal difference between the annotated seizure onset and the first continuous positive prediction produced by the model, operating on non-overlapping 0.5 s EEG windows. Within the dataset, the maximum observed detection delay was 13 s, which was taken as the upper bound for acceptable latency.

Considering the importance of latency alongside accuracy and sensitivity, the overall performance of the proposed model was evaluated with respect to these three factors, as formalized in Equation (1).

(1) $$Score=\alpha \times Acc+\beta \times \left(1-\frac{DD}{13}\right)+\gamma \times (1-\frac{FN}{TS})$$

The score ranged from 0 to 10, with higher values indicating better performances. Detection delay (DD) was normalized by a factor of 13, which corresponded to the maximum delay observed in our experiments. However, as stated earlier, the maximum acceptable detection delay for this system was 7.41 s. Any detection with a delay exceeding 7.41 s was considered suboptimal, leading to a significantly lower score. While delays above this threshold were still recorded for analysis, they indicated a failure in meeting the near-real-time requirements and negatively impacted the model’s overall evaluation. To ensure that no seizure went undetected, minimizing false negatives (FN) was critical. This was achieved by maximizing sensitivity (recall), which directly impacted the model’s reliability. The overall score was computed by weighting three key components: detection delay, accuracy, and false negatives using the parameters alpha (α), beta (β), and gamma (γ), based on the project’s objectives. To analyze the behavior of the proposed composite score under different performance trade-offs, five representative model configurations (M1–M5) were selected, shown in Table 2, each emphasizing a distinct characteristic, including high detection delay, low sensitivity, low accuracy, high sensitivity, and high accuracy. These configurations served as controlled reference points for evaluating the impact of different scoring priorities.

Given that the primary objective of this work was to avoid missed seizures while maintaining low detection latency and high overall accuracy, multiple clinically motivated weighting schemes (W1–W6) were evaluated, as shown in Table 3. These weight sets emphasized different priority hierarchies among sensitivity, detection delay, and accuracy, allowing assessment of score robustness and ranking consistency across reasonable operational scenarios, rather than relying on a single fixed weighting configuration.

**Figure 2:**
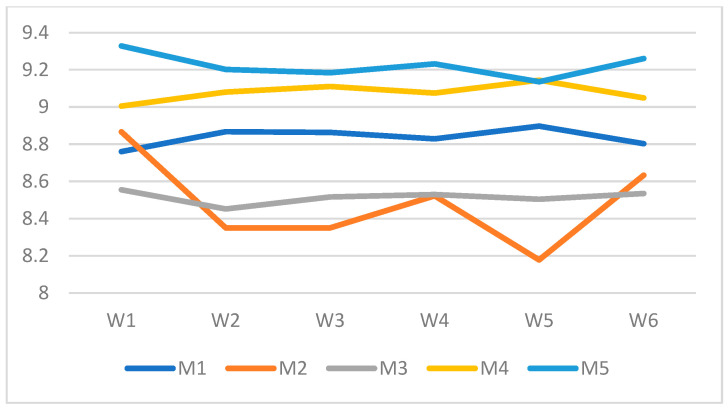
Score performance across control models in different weight distributions.

The weighting configuration W1 was selected as the reference scheme because it provided a balanced yet clinically aligned prioritization of seizure detection performance. As shown in Figure 2, W1 consistently preserved the relative ranking of models across all representative configurations (M1–M5), while favoring models with high sensitivity and low detection latency without disproportionately penalizing accuracy. Importantly, under W1, the high-sensitivity and high-accuracy models (M4 and M5) achieved the highest composite scores, aligning with the primary clinical objective of minimizing missed seizures while maintaining an under-a-second detection delay. This stability across diverse model behaviors indicated that W1 offered a robust and interpretable scoring configuration rather than one tuned to a specific performance extreme. The composite score was intended as an auxiliary indicator to summarize trade-offs among multiple performance criteria and was not used as a standalone or primary metric for model selection.

**Figure 3:**
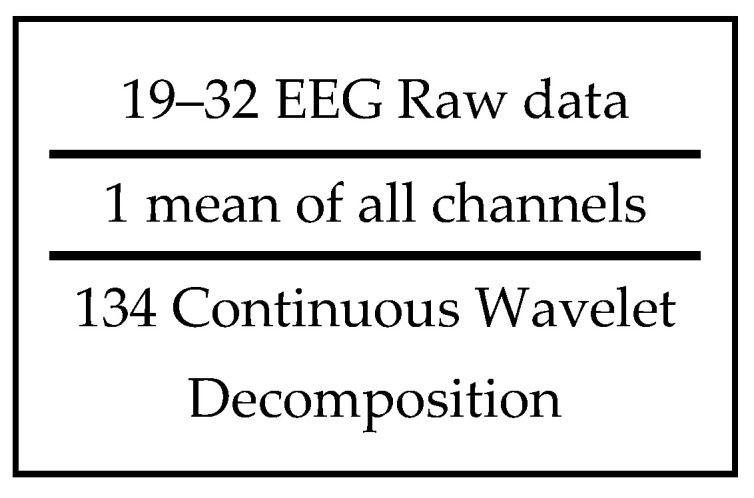
Data frame structure used for model training.

All seizures in the dataset were manually annotated with a temporal precision of 0.01 s relative to their start and end times. These annotations produced a binary sequence aligned with the 19 EEG channels, where a value of 1 indicated seizure activity and 0 indicated non-seizure periods. Because of variations in electrode placement across the scalp, the signals were prone to contamination from sources such as eye blinks and environmental noise. Computing the arithmetic mean of the 19 raw signals could help reduce the impact of such unwanted components. The EEG recordings contained a large amount of information in both the time and frequency domains. To capture additional informative patterns, wavelet transform features were extracted and combined with the raw EEG signals. Class balancing was achieved by roughly selecting equal-duration non-seizure segments for each seizure segment, sampled from interictal periods within the same recordings to avoid temporal overlap with seizure activity. Balanced segments containing both seizure and non-seizure data were then divided into 5 min packets, as illustrated in Figure 3.

Most existing seizure detection systems rely on EEG helmets with more than 32 electrodes. While using a high number of channels can improve detection accuracy, managing extensive electrode setups can be uncomfortable for patients and less suitable for daily use. Reducing the dimensionality of the data also enables deployment on lower-cost wearable devices with limited processing power. To address these issues, the final stage of this research incorporated a feature selection process that reduced the number of raw EEG channels by approximately 70 percent.

The experiments were carried out on a workstation equipped with an Intel i7 9700 CPU (3 GHz), an Asus RTX 2080 Ti GPU, and 32 GB of DDR4 RAM. The recurrent neural network was implemented in MATLAB 2024b. Preprocessing was executed on a single CPU core, and model training was accelerated using the GPU, whereas inference was performed entirely on the CPU. The reported response time corresponded to the duration required to classify a 0.5 s EEG data frame. Maintaining this response time below 0.1 s was essential for near-real-time operation, and further reduction is desirable to support deployment on less powerful computing platforms.

## 4. Results and Analysis

### 4.1. Overview of Experimental Setup and Data Partitioning

The experimental evaluation was designed to assess the performance of the proposed BiLSTM-based seizure detection framework using the EPILEPSIAE dataset. All experiments were conducted using pre-segmented EEG recordings consisting of balanced seizure and non-seizure intervals, compiled into five-minute data packets. Due to the extreme class imbalance inherent in continuous EEG recordings, seizure detection models trained on fully realistic data distributions often converge toward trivial solutions that favor non-seizure predictions, resulting in excessively high false-negative rates. To mitigate this bias and enable stable training and comparative evaluation of model architectures, seizure and non-seizure segments were artificially balanced in this study.

This design choice allowed the proposed framework to be evaluated in terms of its intrinsic detection capability rather than its sensitivity to class prevalence. However, it does not reflect the true operating conditions of long-term clinical monitoring, where seizure events are rare. Consequently, the reported performance metrics should not be interpreted as direct estimates of real-world false alarm rates.

The dataset was partitioned at the seizure level rather than the patient level, such that at least one seizure from each patient was included in the training set. The experimental protocol allowed seizures from the same patient to appear in both training and test sets. Consequently, the reported results reflect patient-inclusive performance rather than patient-independent generalization.

This evaluation protocol was adopted to ensure sufficient exposure of the model to diverse seizure morphologies during training and facilitate stable model convergence on the large-scale EPILEPSIAE dataset. However, this design does not constitute a fully patient-independent evaluation, and, therefore, the reported results should be interpreted as reflecting within-patient and cross-seizure generalization rather than strict generalization to unseen patients. A more detailed investigation of inter- and intra-patient variability, including patient-wise validation and per-patient statistics, remains an important direction for future work. Further, all reported performance metrics were obtained using Monte Carlo cross-validation with five independent shuffled train-test splits. The final results are reported as the means and standard deviations across these runs.

Each seizure was manually annotated with a temporal precision of 0.01 s and the binary seizure labels were synchronized with 19 EEG channels. In addition to the raw EEG signals, wavelet transform features were included to enhance the feature space and improve model robustness. The evaluation focused on three main aspects:

1. Generalized Model Performance: Establishing baseline seizure detection results using the BiLSTM model across different subsets of the EPILEPSIAE dataset.
2. Optimization and Reduction Strategies: Investigating the impact of hyperparameter tuning and electrode reduction on model performance, with the goal of enabling efficient and potentially wearable near-real-time applications.
3. Comparative and Adaptive Analysis: Validating the generalizability of the model by testing on the MIT dataset and examining the benefits of outlier seizure retraining for improving recall and reducing detection delay.

Performance was evaluated using multiple metrics to provide a comprehensive assessment of the detection system. Accuracy was used to measure overall classification performance. Recall (sensitivity) was prioritized as the primary clinical metric, reflecting the ability to correctly identify seizures. Detection delay quantified the time difference between seizure onset and correct model prediction. These metrics were chosen to capture both the algorithmic accuracy and the clinical relevance of the system. In addition, the score, defined in Equation (1), was applied to combine these metrics into a single performance indicator, enabling a balanced assessment of both algorithmic accuracy and near-real-time clinical relevance.

### 4.2. Performance on Individual Subsets

The individual patient data was extracted according to Figure 3, with data framed and appended with the wavelet decomposition. The resulting data frame contained 19 raw EEG data alongside an arithmetic mean and a total of 134 additional wavelet features.

The initial phase of the experimentation, detailed in Table 4, involved a systematic evaluation of model architecture and hyperparameters across four distinct patient subsets. This analysis aimed to identify an optimal configuration by comparing a single BiLSTM layer with 100 hidden nodes versus one with 200 hidden nodes, while also tuning the learning rate. A clear trend emerged, demonstrating that the more complex model with 200 hidden nodes, when paired with a smaller learning rate of 0.01, consistently yielded superior performance. This was best exemplified by the Coimbra subset, where the 200-node model achieved the highest overall score of 8.887, with zero undetected seizures and a strong recall of 64.61%. This establishes that a model with greater network capacity is better equipped to learn the intricate patterns of seizure activity.

This comparative analysis also underscored the critical importance of our custom Score metric for a balanced and clinically relevant assessment. The results revealed instances, such as within the Co Surf subset, where a 200-node model with a high learning rate achieved an exceptional recall of 96.78% at the cost of poor accuracy of 40.79%, indicating a high rate of false positives. Relying on a single metric would have been misleading; however, our composite score effectively penalized such imbalanced outcomes and identified models that optimally balanced accuracy, recall, and detection latency. This holistic evaluation framework is essential for navigating the classic trade-off between model sensitivity and specificity, especially when employing more complex architectures.

Finally, the significant performance variability observed across the different patient subsets highlights the profound challenge posed by inter-patient heterogeneity. While the optimized 200-node model successfully eliminated all undetected seizures for the Surf 30 and Coimbra sets, the PA surf subset proved more difficult, suggesting the presence of more irregular seizure patterns. This finding validates the diversity within the EPILEPSIAE dataset and reinforces the necessity of selecting a sufficiently complex architecture before training a generalized model. The insights from this architectural tuning provide a crucial, data-driven foundation for developing a unified model capable of performing reliably across this wide spectrum of patient data. A fully patient-wise evaluation, in which all seizures from a given patient are excluded from the training set, was not conducted in this study. Future work will focus on patient-independent validation to further assess generalization to unseen subjects.

### 4.3. Overall Performance Across the Dataset

The total extracted raw EEG data contained 1032 epileptic seizures ranging from 6 to 140 s long, averaging at about 60 s. The EEG data was sampled at 256 Hz. However, since the placement of the EEG electrodes was different in some patients, all rows of data were rearranged and the common 19 probe positions were extracted.

Following the subset-specific tuning, a generalized model was evaluated on the entire combined dataset, with the results presented in Table 5. This comprehensive analysis aimed to identify the single, optimal model configuration capable of performing robustly across the diverse 161-patient cohort. The findings clearly indicate that a BiLSTM network with 200 hidden nodes, trained with a learning rate of 0.01, provides the best overall performance. This best-performing configuration achieved the highest score of 8.497, driven by a recall of 79.27%, a low detection delay of 1.52 s, and a single completely undetected seizure, establishing it as the most clinically effective and balanced configuration.

The analysis further revealed a distinct optimum in model complexity. While increasing the network from 100 to 200 hidden nodes dramatically improved performance by allowing the model to capture the intricate patterns in the diverse dataset, a further increase to 250 nodes resulted in a decline in both recall and the overall score. This demonstrates that the 200-node architecture provides the optimal capacity for generalization without beginning to overfit the training data. Similarly, a smaller learning rate of 0.01 proved essential for learning robust features, as the larger rate of 0.1 led to a model with critically poor sensitivity. Crucially, the technical feasibility of this approach for near-real-time application was unequivocally validated. Across all tested configurations, the computational time required to classify a 0.5 s EEG window remained consistently low, approximately 0.1 s on a single-core CPU. This confirms that the model is not only clinically effective but also computationally efficient, meeting the stringent low-latency requirements for a system designed to provide proactive, near-real-time seizure detection and intervention.

### 4.4. Electrode Reduction and Feature Selection

Although the results of the experiment validated a relatively reliable detection system, capturing data from all 19 electrodes could be challenging and uncomfortable for the patients. Therefore, we aimed to minimize the number of electrodes required to ensure a more comforting system that could still achieve a standard and reliable seizure detection. The electrode positions and availability among the selected patients from each set were not identical. Therefore, some patients could have 32 EEG inputs while some had only 19. The initial selection approach trimmed the excess EEG features and selected only the common electrodes, which left 15 unique EEG channels. To improve the practicality of our seizure detection framework and reduce computational load, we evaluated reduced electrode configurations. The selection of electrodes was guided by a multi-step, data-driven process:

1. Seizure Origin Analysis: We performed an in-depth analysis of seizure onset locations across all patients to identify regions that contributed most frequently to seizure activity.
2. Correlation Analysis: For each electrode, we computed correlations between input signals and seizure labels to quantify predictive relevance.
3. Shapley Value Assessment: We further evaluated channel importance using Shapley values, which captured the contribution of each electrode to model predictions in a principled, model-aware manner.

Combinations 1 to 4 assessed the performance of the network with a limited number of EEG features. Table 6 and Table 7 list the selected EEG channels in each combination and, more importantly, present the performance metrics for the benchmark, the most common positions, and the four combinations.

The results indicate a strong performance for combo 2, where all seizures but one were detected, and the detection delay was under 0.87 s for the dataset. Given the 80% reduction in input EEG data, the slight performance drop is justified. In compensation for that, patients’ comfort was significantly increased as the headband required only 6 EEG electrodes instead of 32.

The primary objective of the feature selection analysis was to identify a minimal set of EEG channels that could maintain high detection performance, thereby enabling the design of a practical and comfortable wearable device. We first established a high-performance baseline using a comprehensive set of 21–24 EEG channels, which consistently yielded scores above 8.78 across all patient subsets. Subsequent experiments evaluated progressively smaller channel sets, revealing a clear trade-off between the number of inputs and model accuracy. This methodical reduction was essential to quantify the performance cost associated with improving the system’s wearability.

The most significant finding of this study emerged from the evaluation of Combo 2, a configuration using just six strategically placed electrodes (FP1, FP2, F7, O1, F3, FZ). The selected electrodes predominantly covered frontal and temporal regions, which are commonly associated with seizure onset and propagation in focal and generalized epilepsy, consistent with the 10–20 system. This minimalist setup achieved a performance score that was remarkably close to the original baseline, representing only a marginal decrease in overall efficacy despite an 80% reduction in input data. This result demonstrates conclusively that a computationally efficient and patient-friendly system is feasible without a significant compromise in its clinical viability, marking a critical step toward real-world deployment.

Furthermore, our analysis revealed the superiority of knowledge-based channel selection strategy over a purely data-driven one. Alternative configurations based on selecting channels with the highest statistical correlation to seizure events (Combos 3 and 4) failed dramatically, with recall rates plummeting to clinically unacceptable levels. This powerful negative result underscores that the spatial distribution of electrodes is critical for capturing the complex, network-level brain activity characteristic of seizures. The success of Combo 2 provides a clear, evidence-based blueprint for the physical design of future wearable seizure detection hardware.

### 4.5. Final Results

Applying the Combo 2 data filter on the dataset reduced the size of the dataset significantly; however, the performance was relatively similar, with a slight decrease in accuracy. A total of 1032 seizures from 161 patients were extracted, preprocessed, and used for training. Table 8 concludes the performance metrics of the dataset on a number of network topologies.

### 4.6. Timing Analysis

**Figure 4:**
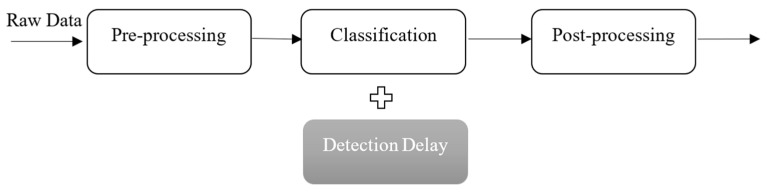
Time analysis stages of the detection system in addition to model introduced detection delay.

Near-real-time seizure detection can prevent escalation of seizures, which can lead to other health problems. The timeline of our seizure detection is shown in Figure 4. Each stage is described below in Table 9.

The data pre-processing stage consumed the most time, while classification and post-processing combined only contributed an insignificant amount of the total detection time. The average end-to-end processing latency was 65 ms per 0.5 s EEG window, satisfying the defined near-real-time constraint. All the timing analysis was performed on a system, with the specifications given in Section 3. However, an additional delay was introduced to the overall detection time, caused by the timing difference between the predicted and labeled seizure. Unlike the other parameters, this factor was directly proportional to the model’s performance and did not rely on any hardware resources.

Although the total time was relatively low, further modifications could be implemented to considerably reduce the time for the preprocessing stage. All timing measurements were obtained on a desktop-class computing platform. No embedded or wearable hardware testing was conducted in this study. Currently, the preprocessing that generates the wavelet features is performed on a single core of the processor. This action can be accelerated through parallelization of the process. Additionally, depending on the model of the GPU and the platform, the entire process can be performed by the GPU.

### 4.7. Comparative Analysis (Compare MIT with EPILAPSIAE)

Another popular dataset in the epilepsy domain is the MIT dataset collected from 23 pediatric patients in the Children’s Hospital Boston [15]. The recordings contain about 10 h of EEG readings at a 256 Hz sampling rate with a 16-bit resolution [15]. The layout of the electrode follows a similar pattern to the one used in the EPILAPSIAE dataset. However, the total number of seizures is limited to 182 seizures.

In our previous work, we proposed a very similar seizure approach process. Initially, the individual seizures are extracted, labeled, and then packetized according to Figure 3. Upon completion of seizure detection, a dimensionality reduction is added to the pipeline with the aim of decreasing the amount of data handled and increasing the comfort of EEG wearables.

The data packets were fed into multiple neural networks of the same structure, shown in Figure 1, with different numbers of hidden layers and nodes. The outcomes were assessed using the score equation, signifying the importance of accuracy and detection delay. The top performing models achieved over 87% for accuracy, with 0 undetected seizures and a total score of 9.24. The detection delay was minimized to 0.51 s on average. The input elimination stage utilized a correlation matrix of the raw input signals against the output. Additionally, the feature importance of the EEG signals was assessed through IBM’s AutoAI. A reduced input model was used for individual and all patients. Table 10 illustrates the impact of the input selection on the performance metrics.

**Figure 5:**
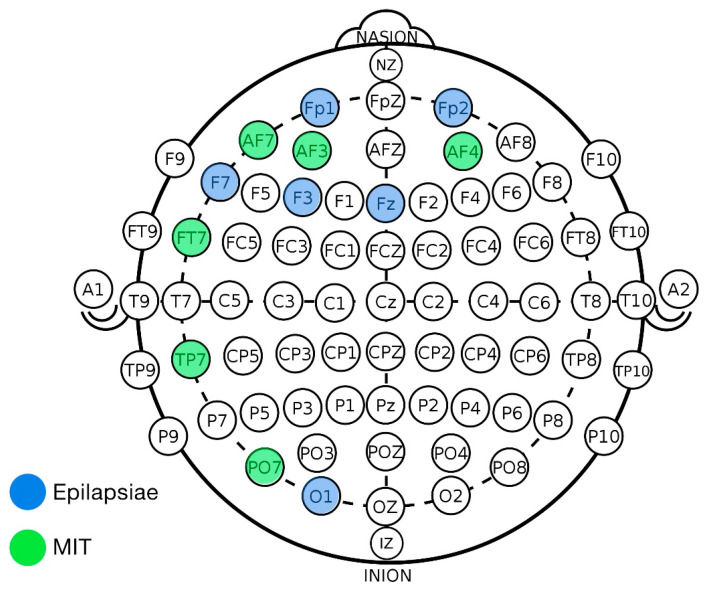
Selected electrodes in the reduced-channel configuration.

**Figure 6:**
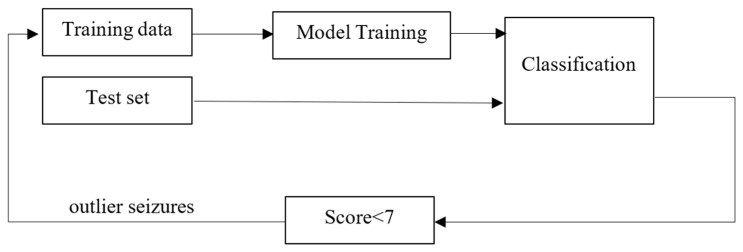
Outlier-aware retraining.

The selected EEG signals were positioned at locations FP1–F7, F7–T7, T7–P7, P7–O1, FP1–F3, and FP2–F4. As shown in Figure 5. Locating the identical signals in the EPILAPSIAE set was challenging as it used a different positioning standard. However, by illustrating them on the scalp, a pattern could be drawn. As indicated in Section 4.3, combination number 2 outperformed the rest of the combinations. Comparing the selected signals of the two datasets, a similar location on the scalp can be found in Figure 6.

It is evident that the frontal section of the brain carries information essential to seizure detection, compared to other locations. Although the performance measures registered lower numbers in recall and detection delay, the extra comfort and lower data manipulation compensated for the deteriorated evaluations.

### 4.8. Exploratory Error-Driven Retraining Analysis

The performance of seizure detection models is strongly affected by seizure morphology and severity, which can vary substantially across patients. While the proposed model achieved satisfactory performance for the majority of seizure events, a small subset of seizures remained consistently undetected. These cases corresponded to seizure episodes for which no true positive prediction was produced during the seizure interval, as reflected by the undetected seizure count reported in Table 2.

In this work, such cases are referred to as outlier seizures. These seizures were identified retrospectively based on low classification performance at the seizure level, rather than sample-level misclassification. The occurrence of outlier seizures suggests that their temporal or spectral characteristics were insufficiently represented in the training data.

To explore whether model exposure to similar seizure patterns can improve detection robustness, an exploratory error-driven retraining procedure was conducted. In this analysis, outlier seizures identified within the training subset were retained and reintroduced during a subsequent retraining phase, forming a closed-loop refinement process, illustrated in Figure 6. Importantly, seizures from the test set were not incorporated into training at any stage.

This analysis is intended as an initial investigation into the potential benefits of targeted retraining for rare or atypical seizure patterns, rather than as a fully developed outlier detection framework. A comprehensive treatment of inter- and intra-patient variability remains an important direction for future work.

## 5. Conclusions

Epilepsy is a neurological disorder marked by recurrent seizures resulting from abnormal brain activity. Accurate and rapid detection of seizures can significantly reduce medical risks and improve the safety and quality of life of patients. This study introduced a near-real-time epileptic seizure detection framework based on bidirectional long short-term memory (BiLSTM) networks combined with wavelet-derived features. The model was trained and evaluated on the large-scale EPILEPSIAE dataset, containing recordings from 161 patients and over 1000 annotated seizures. The proposed BiLSTM-wavelet model demonstrated high detection performance, achieving 86.9% accuracy, 86.1% recall, an average detection delay of 1.05 s, and a total processing time of 0.065 s per 0.5 s EEG window. Importantly, electrode reduction experiments revealed that comparable performance could be maintained using only six EEG electrodes, offering a significant improvement in comfort and practicality for wearable applications. Furthermore, the inclusion of an outlier-aware retraining mechanism improved the detection of irregular seizure patterns and reduced the number of undetected events.

Future work will focus on extending this framework to patient-specific and multimodal systems that integrate additional physiological signals such as ECG and EMG. Further optimization for embedded and mobile hardware will also be pursued to enable continuous, low-power, near-real-time seizure monitoring suitable for clinical and home environments.

## Figures and Tables

**Table 1:** Comparison of representative EEG-based seizure detection studies relevant to this work.

Study	Method	Accuracy %	Delay (s)	Key Contributions
Ullah et al. (2018) [45]	CNN + RNN	95.0	5.2	Achieved high accuracy in automated seizure detection
Chung et al. (2024) [46]	Localization + CNN	96.76–98.66	2.1–3.4	Developed a single-channel CNN-based seizure detection system with clinical confirmation of seizure locations
Shen et al. (2023) [47]	Tunable-Q wavelet transform	97.57	10.46	The proposed algorithm achieved high accuracy (97.57%), sensitivity (98.90%) and a low false positive rate (2.13%) for real-time epilepsy seizure detection. However, the 10.46 s detection delay can be further improved.
Mir et al. (2023) [48]	Bi-LSTM	99.8	4	The proposed DCAE-ESD-Bi-LSTM model achieved very high performance metrics for epileptic seizure detection, including 99.8% accuracy, 99.7% F1 score, and 99.8% sensitivity on the 4 s EEG segment length.
Shen et al. (2022) [49]	Discrete wavelet transform and machine learning methods	96.38	10.42	Developed a real-time EEG-based seizure detection system that emphasizes minimizing detection latency while maintaining acceptable accuracy.

**Table 2:** Performance characteristics of representative model configurations (M1–M5) illustrating different trade-offs between accuracy, sensitivity, and detection latency.

Model	Characteristic	Accuracy	Sensitivity	Detection Delay	Undetected Seizure Count
M1	High Delay	0.86299 ± 0.02	0.8124 ± 0.02	3.523 ± 0.85	1
M2	Low Sensitivity	0.86116 ± 0.03	0.7488 ± 0.01	1.031 ± 0.34	1
M3	Low Accuracy	0.75602 ± 0.05	0.7920 ± 0.03	1.002 ± 0.17	0
M4	High Sensitivity	0.88111 ± 0.01	0.9800 ± 0.02	1.003 ± 0.37	0
M5	High Accuracy	0.96048 ± 0.02	0.9101 ± 0.01	0.942 ± 0.12	0

**Table 3:** Composite score evaluation of models M1–M5 under different clinically motivated weighting schemes (W1–W6).

Weights	Accuracy α	Sensitivity β	Delay γ	Interpretation
W1	0.4	0.4	0.2	Original baseline
W2	0.3	0.5	0.2	Sensitivity-prioritized
W3	0.25	0.5	0.25	Safety-critical-balanced
W4	0.3	0.4	0.3	Latency-aware
W5	0.2	0.6	0.2	Extreme safety focus
W6	0.33	0.33	0.34	Neutral reference

**Table 4:** Performance across individual feature subsets.

Set	Total Features	Model	Accuracy	Number of Seizures	Undetected Seizures	Detection Delay (s)	Recall	Score
Surf_30	154	200 h with LR 0.01	77.76 ± 2.11%	196	0	1.28 ± 0.31	58.43 ± 5.97%	8.51
Co Surf	154	100 h with LR 0.1	80.64 ± 1.42%	72	0	0.55 ± 0.03	48.58 ± 4.16%	8.71
Coimbra	154	200 h with LR 0.01	82.03 ± 1.85%	302	0	0.66 ± 0.11	64.61 ± 2.84%	8.88
PA surf	154	100 h with LR 0.01	81.83 ± 1.06%	383	1	3.00 ± 0.52	45.79 ± 3.13%	8.31

**Table 5:** Performance using all available data from the set.

Model (Hidden Nodes + Learn Rate)	Accuracy	Undetected Seizures	Detection Delay	Recall	Score
100 h + 0.1	0.780 ± 0.04	56	12.96 ± 2.4	0.034 ± 0.02	6.27
100 h + 0.01	0.821 ± 0.02	28	6.87 ± 1.63	0.275 ± 0.03	7.58
200 h + 0.1	0.776 ± 0.02	2	2.34 ± 1.04	0.293 ± 0.08	8.12
200 h + 0.01	0.849 ± 0.03	1	1.526 ± 0.62	0.792 ± 0.04	8.49
200 h + 100 h + 0.01	0.783 ± 0.02	14	4.56 ± 0.71	0.445 ± 0.04	7.94
250 h + 0.01	0.790 ± 0.02	3	2.73 ± 0.31	0.499 ± 0.04	8.30

**Table 6:** Performance of selected feature combinations.

Sets	Inputs	Total Features	Accuracy	Undetected Seizures	Detection Delay	Recall	Score
Common electrode baseline (19–24 channels)	Raw EEG (19–24) + mean + wavelet	154–159	80.57 ± 2.3%	1	1.38 ± 0.7	71.55 ± 3.7%	8.22
Most common positions (15 electrodes)	Raw EEG (15) + mean + wavelet	150	82.80 ± 3.1%	0	1.02 ± 0.3	71.57 ± 6.1%	8.42
Combo 1	Raw EEG (5) + mean + wavelet	140	81.81 ± 5.5%	1	1.68 ± 0.6	65.32 ± 4.5%	8.05
Combo 2	Raw EEG (6) + mean + wavelet	141	86.22 ± 2.6%	1	0.87 ± 0.2	80.40 ± 3.5%	8.78
Combo 3	Raw EEG (4) + mean + wavelet	139	80.03 ± 3.1%	5	1.46 ± 0.3	74.82 ± 2.7%	8.24
Combo 4	Raw EEG (6) + mean + wavelet	141	79.95 ± 2.2%	3	1.49 ± 0.1	78.39 ± 1.9%	8.30

**Table 7:** Final selected feature combination.

Name	Justification	Selected Features
Common 15	Most commonly used positions	C3, C4, CZ, F3, F4, F7, F8, FP1, FP2, FZ, O1, O2, P3, P4, PZ
Combo 1	Similarity-based approach	FP1, FP2, F7, O1, F3
Combo 2	Similarity-based approach	FP1, FP2, F7, O1, F3, FZ
Combo 3	Top 4 based on correlation	O2, O1, F7, PZ
Combo 4	Top 6 based on correlation	O2, O1, F7, PZ, C3, CZ

**Table 8:** Performance across various network architectures.

Hidden Nodes	LR	Accuracy	Undetected Seizures	Detection Delay	Recall	Score
200	0.01	86.91 ± 1.4%	0	1.04 ± 0.10	86.14 ± 1.8%	8.87
100	0.01	82.23 ± 1.2%	0	1.00 ± 0.04	81.66 ± 2.8%	8.61
200	0.01	78.04 ± 2.8%	1	0.94 ± 0.01	87.19 ± 2.5%	8.57
100	0.001	83.72 ± 1.1%	1	1.12 ± 0.02	76.15 ± 4.5%	8.52
150	0.01	81.97 ± 2.8%	0	1.02 ± 0.02	77.16 ± 3.8%	8.50
100	0.0001	82.65 ± 1.2%	2	1.34 ± 0.31	78.28 ± 2.5%	8.45
100	0.1	77.71 ± 3.6%	1	1.24 ± 0.3	84.02 ± 2.1%	8.40
200	0.001	82.95 ± 3.1%	2	1.03 ± 0.1	63.26 ± 6.9%	8.26
150	0.01	81.57 ± 2.1%	1	2.00 ± 0.42	76.39 ± 5.1%	8.17
250	0.01	83.05 ± 2.4%	1	3.24 ± 0.49	63.52 ± 3.5%	7.59
300	0.01	79.03 ± 1.4%	0	4.87 ± 0.81	59.12 ± 8.3%	6.84
200 + 100	0.001	83.41 ± 1.9%	0	3.73 ± 0.77	58.22 ± 5.2%	7.35
100 + 100	0.001	84.92 ± 2.1%	2	2.41 ± 0.62	71.16 ± 2.5%	8.07
150 + 100	0.001	83.15 ± 2.3%	5	4.15 ± 3.2	73.34 ± 3.2%	7.51
200 + 100 + 50	0.0001	80.91 ± 1.5%	12	7.31 ± 1.4	67 ± 4.8%	6.31

**Table 9:** Time duration of each processing stage.

Name	Description	Time (s)
Pre-processing	The time it takes to capture all raw data for 0.5 s, generate mean and wavelet data, and format them into the data structure	0.01779 ± 0.005
Classification on CPU	The time that the neural network takes to classify a 0.5 s window of the data structure on a single CPU (also referred to as response time)	0.0425323 ± 0.003
Classification on GPU	The time that the neural network takes to classify a 0.5 s window of the data structure on a GPU	0.001241 ± 0.0002
Post-processing	Converting neural network output to meaningful labels	0.00471 ± 0.00005
Total	Total time taken from data capture until the end of detection	0.065

**Table 10:** Effect of channel reduction on performance.

Description	Number of EEG Signals	Accuracy	Detection Delay	Recall	Score
Original	19	84.94 ± 0.02	1.523 ± 0.4	79.27 ± 3.2%	8.49
Limited input	6	86.91 ± 0.03	1.048 ± 0.3	86.13 ± 2.0%	8.87

## Data Availability

No new data were created or analyzed in this study. Data sharing is not applicable to this article.

## Abbreviations

The following abbreviations are used in this manuscript:

ANN	Artificial Neural Network
Bi-LSTM	Bi-Directional Long Short-Term Memory
CNNs	Convolutional Neural Network
ECG	Electrocardiogram
EEG	Electroencephalogram
EMG	Electromyography
HUC	Hospitais da Universidade de Coimbra
iEEG	Intracranial EEG
KNN	K-Nearest Neighbor
LSTM	Long Short-Term Memory
RNN	Recurrent Neural Network
SVM	Support Vector Machine
UKLFR	Universitätsklinikum Freiburg

## References

[1] World Health Organization (2021). Epilepsy Facts.

[2] Moshé S.L., Perucca E., Ryvlin P., Tomson T. (2015). Epilepsy: New Advances. Lancet.

[3] Ramgopal S., Thome-Souza S., Jackson M., Kadish N.E., Fernández I.S., Klehm J., Loddenkemper T. (2014). Seizure Detection, Seizure Prediction, and Closed-Loop Warning Systems in Epilepsy. Epilepsy Behav..

[4] Vandecasteele K., De Cooman T., Gu Y., Cleeren E., Claes K., Paesschen W.V., Huffel S.V., Hunyadi B. (2017). Automated Epileptic Seizure Detection Based on Wearable ECG and PPG in a Hospital Environment. Sensors.

[5] Hasan T.F., Tatum W.O. (2021). Ambulatory EEG Usefulness in Epilepsy Management. J. Clin. Neurophysiol..

[6] Beghi E. (2020). The Epidemiology of Epilepsy. Neuroepidemiology.

[7] Current Opinion in Neurology. https://journals.lww.com/co-neurology/Abstract/2003/04000/Do_epileptic_seizures_damage_th%20e_brain_.12.aspx.

[8] Amin U., Nascimento F.A., Karakis I., Schomer D., Benbadis S.R. (2023). Normal Variants and Artifacts: Importance in EEG Interpretation. Epileptic Disord..

[9] Kälviäinen R., Reinikainen M. (2019). Management of Prolonged Epileptic Seizures and Status Epilepticus in Palliative Care Patients. Epilepsy Behav..

[10] Mporas I., Tsirka V., Zacharaki E.I., Koutroumanidis M., Richardson M., Megalooikonomou V. (2015). Seizure Detection Using EEG and ECG Signals for Computer-Based Monitoring, Analysis and Management of Epileptic Patients. Expert Syst. Appl..

[11] Shoeibi A., Khodatars M., Ghassemi N., Jafari M., Moridian P., Alizadehsani R., Panahiazar M., Khozeimeh F., Zare A., Hosseini-Nejad H. (2021). Epileptic Seizures Detection Using Deep Learning Techniques: A Review. Int. J. Environ. Res. Public Health.

[12] Afsari K., El Barachi M., Fasciani S., Belqasmi F. (2022). A Deep Learning Approach for Real-Time Detection of Epileptic Seizures Using EEG. Proceedings of the 2022 7th International Conference on Smart and Sustainable Technologies (SpliTech).

[13] Dash D.P., Kolekar M., Chakraborty C., Khosravi M.R. (2024). Review of Machine and Deep Learning Techniques in Epileptic Seizure Detection Using Physiological Signals and Sentiment Analysis. ACM Trans. Asian Low Resour. Lang. Inf. Process..

[14] Ihle M., Feldwisch-Drentrup H., Teixeira C.A., Witon A., Schelter B., Timmer J., Schulze-Bonhage A. (2012). Epilepsiae—A European Epilepsy Database. Comput. Methods Programs Biomed..

[15] Shoeb A. CHB-MIT Scalp EEG Database 2010.

[16] Monitoring Brain Activity in VR: EEG and Neuroimaging. https://link.springer.com/chapter/10.1007/7854_2023_423.

[17] Constant I., Sabourdin N. (2012). The EEG Signal: A Window on the Cortical Brain Activity. Pediatr. Anesth..

[18] Andrade-Machado R., Cuartas V.B., Muhammad I.K. (2021). Recognition of Interictal Ictal Discharges on, E.E.G. Focal vs Generalized Epilepsy. Epilepsy Behav..

[19] Advances in Electrode Materials for Scalp, Forehead, and Ear EEG: A Mini-Review. https://pubs.acs.org/doi/abs/10.1021/acsabm.3c00322.

[20] Mercier M.R., Dubarry A.-S., Tadel F., Avanzini P., Axmacher N., Cellier D., Vecchio M.D., Hamilton L.S., Hermes D., Kahana M.J. (2022). Advances in Human Intracranial Electroencephalography Research, Guidelines and Good Practices. NeuroImage.

[21] Homan R.W., Herman J., Purdy P. (1987). Cerebral Location of International 10–20 System Electrode Placement. Electroencephalogr. Clin. Neurophysiol..

[22] Alsuwaiket M.A. (2022). Feature Extraction of EEG Signals for Seizure Detection Using Machine Learning Algorthims. Eng. Technol. Appl. Sci. Res..

[23] Sharmila A., Geethanjali P. (2020). Evaluation of Time Domain Features on Detection of Epileptic Seizure from EEG Signals. Health Technol..

[24] Harpale V.K., Bairagi V.K. Time and Frequency Domain Analysis of EEG Signals for Seizure Detection: A Review. Proceedings of the 2016 International Conference on Microelectronics, Computing and Communications (MicroCom).

[25] Wang L., Xue W., Li Y., Luo M., Huang J., Cui W., Huang C. (2017). Automatic Epileptic Seizure Detection in EEG Signals Using Multi-Domain Feature Extraction and Nonlinear Analysis. Entropy.

[26] Van Esbroeck A., Smith L., Syed Z., Singh S., Karam Z. (2016). Multi-Task Seizure Detection: Addressing Intra-Patient Variation in Seizure Morphologies. Mach. Learn..

[27] Kim T., Nguyen P., Pham N., Bui N., Truong H., Ha S., Vu T. (2020). Epileptic Seizure Detection and Experimental Treatment: A Review. Front. Neurol..

[28] Maheshwari J., Joshi S.D., Gandhi T.K. (2022). Real-Time Automated Epileptic Seizure Detection by Analyzing Time-Varying High Spatial Frequency Oscillations. IEEE Trans. Instrum. Meas..

[29] Assali I., Ghazi Blaiech A., Ben Abdallah A., Ben Khalifa K., Carrère M., Hédi Bedoui M. (2023). CNN-Based Classification of Epileptic States for Seizure Prediction Using Combined Temporal and Spectral Features. Biomed. Signal Process. Control.

[30] Lerogeron H., Picot-Clémente R., Rakotomamonjy A., Heutte L. (2023). Approximating Dynamic Time Warping with a Convolutional Neural Network on EEG Data. Pattern Recognit. Lett..

[31] Abdelhameed A.M., Daoud H.G., Bayoumi M. Deep Convolutional Bidirectional LSTM Recurrent Neural Network for Epileptic Seizure Detection. Proceedings of the 2018 16th IEEE International New Circuits and Systems Conference (NEWCAS).

[32] Tang Y., Wu Q., Mao H., Guo L. (2024). Epileptic Seizure Detection Based on Path Signature and Bi-LSTM Network with Attention Mechanism. IEEE Trans. Neural Syst. Rehabil. Eng..

[33] Tuncer E., Doğru Bolat E. (2022). Classification of Epileptic Seizures from Electroencephalogram (EEG) Data Using Bidirectional Short-Term Memory (Bi-LSTM) Network Architecture. Biomed. Signal Process. Control.

[34] Cao X., Zheng S., Zhang J., Chen W., Du G. (2025). A Hybrid CNN-Bi-LSTM Model with Feature Fusion for Accurate Epilepsy Seizure Detection. BMC Med. Inf. Decis. Mak..

[35] Kashefi Amiri H., Zarei M., Daliri M.R. (2025). Epileptic Seizure Detection from Electroencephalogram Signals Based on 1D CNN-LSTM Deep Learning Model Using Discrete Wavelet Transform. Sci. Rep..

[36] Majzoub S., Fahmy A., Sibai F., Diab M., Mahmoud S. (2023). Epilepsy Detection with Multi-Channel EEG Signals Utilizing AlexNet. Circuits Syst. Signal Process..

[37] Raibag M.A., Rashmi B.C., Gorikhan I.A., Sarkar R., Deka N. A Deep Learning and Raspberry PI Investigation for Epilepsy Seizure Detection. Proceedings of the 2024 Third International Conference on Artificial Intelligence, Computational Electronics and Communication System (AICECS).

[38] Maher C., Yang Y., Truong N.D., Wang C., Nikpour A., Kavehei O. (2023). Seizure Detection with Reduced Electroencephalogram Channels: Research Trends and Outlook. R. Soc. Open Sci..

[39] Zubair M., Belykh M.V., Naik M.U.K., Gouher M.F.M., Vishwakarma S., Ahamed S.R., Kongara R. (2021). Detection of Epileptic Seizures from EEG Signals by Combining Dimensionality Reduction Algorithms with Machine Learning Models. IEEE Sens. J..

[40] Razi K.F., Schmid A. (2022). Epileptic Seizure Detection with Patient-Specific Feature and Channel Selection for Low-Power Applications. IEEE Trans. Biomed. Circuits Syst..

[41] Liu X., Richardson A.G. (2021). Edge Deep Learning for Neural Implants: A Case Study of Seizure Detection and Prediction. J. Neural Eng..

[42] Idrees A.K., Idrees S.K., Couturier R., Ali-Yahiya T. (2022). An Edge-Fog Computing-Enabled Lossless EEG Data Compression with Epileptic Seizure Detection in IoMT Networks. IEEE Internet Things J..

[43] Sayeed M.A., Mohanty S.P., Kougianos E., Yanambaka V.P., Zaveri H. A Robust and Fast Seizure Detector for IoT Edge. Proceedings of the 2018 IEEE 4th International Symposium on Smart Electronic Systems (iSES).

[44] Aldahr R.S., Alanazi M., Ilyas M. Addressing Inter-Patient Variability in EEG: Diversity-Enhanced Data Augmentation and Few-Shot Learning-Based Epilepsy Detection. Proceedings of the 2022 International Conference on Healthcare Engineering (ICHE).

[45] Ullah I., Hussain M., Qazi E.-H., Aboalsamh H. (2018). An Automated System for Epilepsy Detection Using EEG Brain Signals Based on Deep Learning Approach. Expert Syst. Appl..

[46] Chung Y.G., Cho A., Kim H., Kim K.J. (2024). Single-Channel Seizure Detection with Clinical Confirmation of Seizure Locations Using CHB-MIT Dataset. Front. Neurol..

[47] Shen M., Wen P., Song B., Li Y. (2023). Real-Time Epilepsy Seizure Detection Based on EEG Using Tunable-Q Wavelet Transform and Convolutional Neural Network. Biomed. Signal Process. Control.

[48] Mir W.A., Anjum M., Izharuddin I., Shahab S. (2023). Deep-EEG: An Optimized and Robust Framework and Method for EEG-Based Diagnosis of Epileptic Seizure. Diagnostics.

[49] Shen M., Wen P., Song B., Li Y. (2022). An EEG based real-time epilepsy seizure detection approach using discrete wavelet transform and machine learning methods. Biomed. Signal Process. Control.

[50] Zhang Y., Yao S., Yang R., Liu X., Qiu W., Han L., Zhou W., Shang W. (2022). Epileptic Seizure Detection Based on Bidirectional Gated Recurrent Unit Network. IEEE Trans. Neural Syst. Rehabil. Eng..

[51] Kim M., Jo D., Wang I.-N., Kim H., Kim J.B., Kim D.-J. Enhancing Everyday Seizure Detection: A Channel Reduction Approach. Proceedings of the 2024 12th International Winter Conference on Brain-Computer Interface (BCI).

[52] Ahmadi M.B., Craik A., Azgomi H.F., Francis J.T., Contreras-Vidal J.L., Faghih R.T. (2019). Real-Time Seizure State Tracking Using Two Channels: A Mixed-Filter Approach. Proceedings of the Conference Record—53rd Asilomar Conference on Circuits, Systems and Computers, ACSSC 2019.

[53] Böttcher S., Bruno E., Epitashvili N., Dümpelmann M., Zabler N., Glasstetter M., Ticcinelli V., Thorpe S., Lees S., Van Laerhoven K. (2022). Intra- and Inter-Subject Perspectives on the Detection of Focal Onset Motor Seizures in Epilepsy Patients. Sensors.

